# ROCKETSHIP: a flexible and modular software tool for the planning, processing and analysis of dynamic MRI studies

**DOI:** 10.1186/s12880-015-0062-3

**Published:** 2015-06-16

**Authors:** Samuel R. Barnes, Thomas S. C. Ng, Naomi Santa-Maria, Axel Montagne, Berislav V. Zlokovic, Russell E. Jacobs

**Affiliations:** 1 Division of Biology and Biological Engineering, California Institute of Technology, Pasadena, CA 91125 USA; 2 Department of Medicine, University of California, Irvine Medical Center, Orange, CA USA; 3 Zilkha Neurogenetic Institute and Department of Physiology and Biophysics, Keck School of Medicine, University of Southern California, Los Angeles, CA USA

**Keywords:** DCE-MRI, Imaging, ADC, Parametric mapping, Data-driven kinetic modelling, MATLAB, MRI, Nested model

## Abstract

**Background:**

Dynamic contrast-enhanced magnetic resonance imaging (DCE-MRI) is a promising technique to characterize pathology and evaluate treatment response. However, analysis of DCE-MRI data is complex and benefits from concurrent analysis of multiple kinetic models and parameters. Few software tools are currently available that specifically focuses on DCE-MRI analysis with multiple kinetic models. Here, we developed ROCKETSHIP, an open-source, flexible and modular software for DCE-MRI analysis. ROCKETSHIP incorporates analyses with multiple kinetic models, including data-driven nested model analysis.

**Results:**

ROCKETSHIP was implemented using the MATLAB programming language. Robustness of the software to provide reliable fits using multiple kinetic models is demonstrated using simulated data. Simulations also demonstrate the utility of the data-driven nested model analysis. Applicability of ROCKETSHIP for both preclinical and clinical studies is shown using DCE-MRI studies of the human brain and a murine tumor model.

**Conclusion:**

A DCE-MRI software suite was implemented and tested using simulations. Its applicability to both preclinical and clinical datasets is shown. ROCKETSHIP was designed to be easily accessible for the beginner, but flexible enough for changes or additions to be made by the advanced user as well. The availability of a flexible analysis tool will aid future studies using DCE-MRI.

A public release of ROCKETSHIP is available at https://github.com/petmri/ROCKETSHIP.

## Background

The utility of dynamic magnetic resonance imaging (MRI) studies to diagnose diseases, characterize their progression, and evaluate treatment response is a topic of vigorous research. In particular, physiological information garnered from techniques such as diffusion MRI, blood oxygen-level dependent (BOLD) MRI, iron-oxide imaging, dynamic susceptibility MRI (DSC-MRI) and dynamic contrast-enhanced MRI (DCE-MRI) [1] are being explored as imaging biomarkers in virtually all aspects of medicine, especially in oncology and neurological diseases.

DCE-MRI involves the rapid serial acquisition of T1-weighted images before, during and after intravenous injection of a contrast agent (CA). The physiological properties of the tissue of interest are inferred by analyzing the image signal change kinetics induced by the CA within the tissue region of interest (ROI). Given the inherent leakiness of tumor blood vessels (which enhances the signal observed at the tumor site due to more CA leakage) [2], the majority of DCE-MRI studies have focused on oncology applications [3]. Studies have especially focused on using DCE-MRI to characterize tumor phenotypes or to evaluate the response of tumors to therapy [4]. The use of DCE-MRI has also been explored in other fields such as obstetrics [5] and the neurosciences [6, 7].

Although several individual studies have demonstrated the promise of DCE-MRI as an imaging biomarker, widespread clinical adoption of this technique remains limited. This is in part because implementation and standardization of DCE-MRI is challenging. Differences in hardware such as the MRI field strength, coil setups and sequence types available at different institutions can affect the quality of the images acquired and constrain the choice of certain parameters such as the volume of interest, spatial and temporal resolution. The choice of contrast agent, each with unique pharmacokinetic properties and thus behavior in vivo [8], also vary greatly between studies. These factors can significantly affect the quality of the parametric maps generated from imaging data [9].

Widespread variability also exists in the processing and analysis of DCE-MRI data. Both semi-quantitative [10, 11] and quantitative parameters [12] are used to analyze the acquired signal intensity time curves. Several studies have demonstrated the utility of either type of parameters to characterize tumor tissues or monitor treatment response [13, 14]. However, challenges remain in determining the specificity of different parameters to the underlying physiology, development of robust quantification techniques for quantitative model fitting (such as the assignment of a robust arterial input function [15] or T1 measurement of tissues [16]), and the selection of appropriate equations or models to fit the acquired data [17].

Recent studies have sought to address and understand these issues. For example, both Cramer et al. [18] and Montagne et al. [6] demonstrated that the Patlak method may accurately estimate the vascular permeability in the intact or near intact blood brain barrier (BBB); a challenge given the low permeability values being considered (K_trans_ < 0.3 mL/100 g/min) [19–21]. In particular, Montagne et al. were able to demonstrate significant differences in permeability within grey and white matter between human subjects with no cognitive impairment, mild cognitive impairment, and with age [6] using Patlak analysis. Two-compartment models have been shown to be more appropriate at higher permeability [18, 22]. Ewing et al. have explored the use of nested model-selection to determine the appropriate physiological models to fit both preclinical and clinical DCE-MRI data [23, 24], while Jackson et al. recently showed good correlation of semi-quantitative metrics with parameters derived from an extended Tofts model in patients with Type 2 Neurofibromatosis [13]. Importantly, these studies suggest that robust analysis of DCE-MRI data may be disease and organism specific and that a data-driven approach that considers a library of processing and analytical methods may be necessary and appropriate for DCE-MRI analysis [12].

Exploration of these different types of DCE-MRI processing and analyses would be facilitated with standardized software. Software standardization can reduce biases and errors that may arise from the type of processing algorithm used for fitting and enable more consistent comparisons between studies. Moreover, availability of a flexible, easily modifiable and extensible software suite allows the researcher/clinician to examine how various factors discussed above may apply to their specific DCE-MRI study.

To date, numerous software packages are available for DCE-MRI analysis. These include general purpose pharmacokinetic analysis packages such as PMOD (pmod.com), WinSAAM [25], JPKD (pkpd.kmu.edu.tw/jpkd) or SAAMII [26]. These packages are often complex to use, are only commercially available and/or require significant pre-processing of the data to adjust for DCE-MRI usage. DCE-MRI specific software packages also exist, including commercially available packages licensed for clinical use [27]. While these are often user friendly and directly integrated with PACS databases, details about each package’s implementation and certain options involved in the fitting algorithms may be unavailable. This can lead to a wide variability in output results [27]. Other packages available include PMI (https://sites.google.com/site/plaresmedima/), pydcemri (github.com/davidssmith/pydcemri), DCEMRI.jl [28], Jim (Xinapse Systems Ltd), BioMap (maldi-msi.org), PermGUI/PCT [29], Toppcat [30], DCEMRIS4 [31], dcetool (http://thedcetool.com/), dcemri (http://dcemri.sourceforge.net/), DATforDCEMRI [32], DCE@UrLAB [33] and UMMPerfusion [34]. Table 1 provides a summary of the capabilities of these packages. While geared for DCE-MRI, most of them are limited in the range of fitting methods, models and parameters that can be analyzed. A number of the packages (developed using IDL and C) are built around a graphical user interface (GUI) that needs to be recompiled each time a new functionality is implemented or a setting changed, which can make module development and individualized changes non-trivial for a novice user. None of the currently available packages in Table 1 enable recently developed data-driven methods, such as nested-model selection, that analyze the appropriateness of the models and parameters being used and generated [23, 24, 35–37]. To circumvent these constraints, groups often develop in-house software for their studies [38–41], with limited availability for other users. This hinders adoption of these methods for widespread use and testing.

**Table 1:** Comparison of existing DCE-MRI packages*

Software package	Language	Operating system	License	Models included	Fitting	Other MRI-relevant features	Input/output	Location
*dcemriS4*	R	Linux, Windows, Mac OS	BSD	- Standard Kelty- Single-compartment model- Extended Kety	- Non-linear least squares- Bayesian estimation	- motion correction and co-registration - B1 mapping - T1 mapping - AIF fitting - DWI fitting - Pixel processing - Job report for later retreival - Access to R functions	- DICOM - NIFTI - Raw data	http://dcemri.sourceforge.net
*dcetool*	C, Plugin for ClearCanvas	Windows	Proprietary	- Tofts - Adiabatic tissue homogeniety - Thorwarth- Extended Tofts - Semi-quantitative metrics (Slope/AUC)	Proprietary		- DICOM - Raw data	http://thedcetool.com/
*PMI*	IDL	Windows	GNU GPL	- Uptake models - Steady-state - Patlak - Model-free deconvolution - Tofts- Extended Tofts - 2CXM - 2C filtration model for kidney - Dual-inlet models for Liver - Semi-quantitative metrics (Slope/Signal enhancement)	- Non-linear Least squares - Truncated singular value decomposition	- ROI and pixel processing - AIF/time series visualization/editing - Access to IDL functions	- DICOM - Raw data	https://sites.google.com/site/plaresmedima/
*UMMPerfusion*	C, OsiriX plugin	Mac OS	BSD	- Model free deconvolution	- Truncated singular value decomposition	- ROI and pixel processing - Job report for later retreival - AIF/time series visualization/editing	- DICOM	http://ikrsrv1.medma.uni-heidelberg.de/redmine/projects/ummperfusion
*Pydcemri*	Python	Linux, Windows, Mac OS	GNU GPL	- Tofts - Extended Tofts	- Non-linear Least squares	- Access to python functions	- Raw data	https://github.com/welcheb/pydcemri
*DCEMRI.jl*	Julia	Linux, Windows, Mac OS	MIT	- Tofts - Extended Tofts - Plasma only	- Non-linear Least squares	- ROI and pixel processing - T1 mapping - Batch processing - Access to Julia functions	- Matlab data	https://github.com/davidssmith/DCEMRI.jl
*DCE@UrLAB*	IDL	Windows	BSD	- Tofts - Hoffmann - Larsson - Fast exchange limit reference region	- Non-linear Least squares	- ROI and pixel processing - Access to IDL functions	- DICOM - Bruker - Raw data	http://www2.die.upm.es/im/archives/DCEurLAB/
*DATforDCEMRI*	R		Creative Commons	- Tofts - Semi-quantitative metrics (AUC, MRT - mean residence time)	- Numerical deconvolution	- Pixel processing - Access to R functions	- R readable data formats	https://github.com/cran/DATforDCEMRI
*TOPPCAT*	Javascript, ImageJ plugin	Linux, Windows, Mac OS	BSD	- Patlak	- Non-linear Least squares	- T1 mapping - Access to ImageJ functions	- ImageJ readable data formats	https://dblab.duhs.duke.edu/modules/dblabs_topcat/
*Jim*	Java	Linux, Windows, Mac OS	Proprietary	- Tofts - Extended Tofts - One compartment - Fermi - 2CXM - Semi-quantitative metrics (AUC)	Proprietary	- ROI and pixel processing	- DICOM - Analyze - Bruker - Commercial formats - Raw data	http://www.xinapse.com/Manual/index.html
*PermGUI*	Matlab	Windows	Creative Commons	- Patlak		- T1 mapping - ROI and pixel processing	- DICOM - Analyze - NIFTI	http://www.quantilyze.com/permgui/
*BioMap*	IDL	Linux, Windows, Mac OS	Proprietary	- Extended Tofts	- Non-linear Least squares	- T1 fitting	- DICOM - Analyze - TIF/PNG	http://www.maldi-msi.org/
*ROCKETSHIP*	Matlab	Linux, Windows, Mac OS	GNU GPL	- Tofts - Extended Tofts - Fast exchange regime (FXR) - 2CXM - Tissue uptake - Nested-model selection - Patlak - Semi-quantitative metrics (AUC)	- Non-linear Least squares	- T1 mapping - ROI and pixel processing - AIF fitting/import - DWI fitting - Job report for later retreival - AIF/time series visualization/editing - Batch processing - Access to Matlab functions - Model fit comparisons with statistical metrics - Drift correction	- DICOM - Analyze - NIFTI - Raw data - Matlab data	https://github.com/petmri/ROCKETSHIP

*Excludes commerical packages for clinical use, AUC: area under curve, DWI: diffusion-weighted imaging

Here, we describe the development of a software suite, ROCKETSHIP (available for download at https://github.com/petmri/ROCKETSHIP), which is implemented in the widely available MATLAB software environment. ROCKETSHIP is designed to be modular, easily extensible and modifiable, and provides tools to process multiple types of parametric MRI datasets. In particular, ROCKETSHIP focuses on the processing and analysis of DCE-MRI data from both human and animal studies, including T1 map generation and arterial input function (AIF) processing and analyses with a library of common pharmacokinetic modeling methods as well as recently developed statistically driven nested model methods. Implementation of ROCKETSHIP in a commonly used language (MATLAB) for image analysis should allow both novice and advance users to utilize and extend the software for their specific DCE-MRI applications.

We test the robustness of this software tool using simulations and demonstrate the utility of ROCKETSHIP to analyze preclinical and clinical DCE-MRI data from different disease models.

## Implementation

All components of ROCKETSHIP were implemented in the MATLAB environment, specifically MATLAB release 2014a. Detailed description of each component can be found in source code comments and documentation that is accessible at the project’s github page (https://github.com/petmri/ROCKETSHIP). In this section, we describe the general architecture of the software suite, and highlight the key aspects of the software and the algorithms currently implemented in the software.

### Architecture design and GUI description

**Fig. 1:**
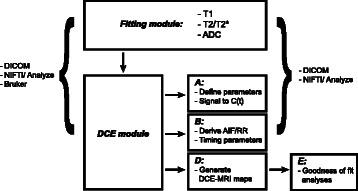
Design outline of ROCKETSHIP. The software suite consists of a fitting module to generate T1, T2/T2* and ADC maps, and DCE-MRI module with sub-modules for each stage of DCE-MRI data processing and analysis

A simplified schematic outlining the design of ROCKETSHIP is shown in Fig. 1. The software is divided into discrete modules to separate processing and analysis components of the pipeline. Initially, a *fitting* module GUI is available for the user to generate voxel-wide parametric maps directly from imaging data. Common MRI parameters such as T2 (and T2*) and T1 relaxation times, and apparent diffusion coefficients (ADC) from diffusion MRI data can be estimated with different fitting methods. A summary of the equations used is in Appendix A. Additional user specified fitting functions can be easily implemented without major modifications to the GUI.

**Fig. 2:**
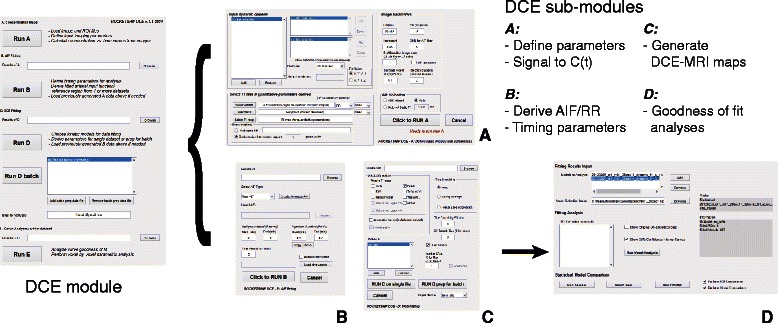
DCE-MRI processing module GUI. GUI modules reflect the schematic outlined in Fig. 2. The “root” DCE module is shown on the left, which launches each sub-module in the pipeline. **a** defines the sub-module that converts raw image data to concentration time curves. The data are passed to the next sub-module, which allows temporal truncation of the dynamic data and fitting or importing of the AIF (**b**). DCE-MRI maps are derived using the next sub-module (**c**). Models can be generated in real time, or the user input can be saved as a data structure job to be run in batch later. Options are provided to perform voxel-by-voxel fits as well as defined ROIs. Raw data curves can be fitted as is, or after being passed through a time smoothing filter. Finally, goodness-of-fit analysis of the fits can be performed with the final sub-module (**d**)

ROCKETSHIP is particularly focused on the processing and analysis of DCE-MRI data. Within the DCE-MRI module (Fig. 2), sub-modules are implemented that focus on:

1. Preparation of the dynamic datasets for DCE-MRI analysis. This involves options for intensity data to concentration versus time curves conversion using T1 maps, selection of the AIF or reference region (RR) and the ROI, noise filtering and signal intensity drift correction over the dynamic time course (Fig. 2a). A summary of how these are implemented is provided in Appendix B.
2. AIF or RR processing with temporal truncation. This sub-module allows the appropriate AIF/RR to be selected. The raw or fitted AIF (currently limited to bi-exponential fitting) from an individual dataset can be used (Fig. 2b). Alternatively, multiple AIFs/RRs can be combined to form a population-averaged AIF/RR. A summary of the implementation is provided in Appendix C. As time duration and temporal resolution can be critical for analysis, options to modify these factors are included.
3. Derivation of DCE-MRI parametric maps (Fig. 2c). Multiple pharmacokinetic models and semi-quantitative metrics can be fitted using ROCKETSHIP. In the current implementation, the Tofts, extended Tofts, Patlak, shutter-speed, two-compartment exchange, tissue uptake models and the area-under-curve (AUC) metric can be calculated (Table 1). Details about these models are summarized in Appendix D. Apart from these stand-alone models, a nested model method is also implemented.

Nesting is based on the hierarchy inherent within the two-compartment exchange model; the algorithm was developed, tested and described in detail by Ewing et al. [12, 23, 24, 37]. First, DCE-MRI data are fitted to the zero-order model, which describes the case where there is insufficient evidence of filling of the vasculature with CA [23, 37]. Next, the initial fit is compared to the data fitting using the one parameter steady-state model:

1 $$ C(t)=v\ {C}_a(t) $$

Where *C*(t) is the concentration of the CA in the voxel of interest at time t, v is the volume fraction of the indicator distribution space and C_a_(t) is the concentration of the CA in the arterial compartment at time t. Here, v can represent the plasma volume fraction (v_p_) in a voxel that is highly vascularized with no vascular/extravascular exchange, the extravascular volume fraction (v_e_) in a weakly vascularized voxel, or v = v_p_ + v_e_ in a fast-exchange scenario. A statistical F-test is used to evaluate the likelihood that an observed improvement of fit to the data, using a model with higher number of parameters, warrants the use of additional parameters [42]. A p-value <0.05 is used as a criterion to increase the number of parameters in the model fit.

In the current implementation, the Patlak model represents the two-parameter form of the two-compartment model with the extended Tofts model with three parameters being at the top of the nested hierarchy [23, 24, 37].

Options to smooth the dynamic signal time course and to fit specific ROIs versus voxel-by-voxel fitting are also available in the DCE-MRI sub-module (Fig. 2c).

4) While models belonging to the same hierarchy can be folded into the nested model fitting option, it may be desirable to compare non-nested models or make comparisons with different statistical tests. The *fitting analysis* sub-module allows for visual and statistical assessment of goodness-of-fit (Fig. 2d). Model fits with 95 % prediction bounds of the fit are shown graphically along with the raw data for each voxel/ROI. Fits between models can be compared using the F-test [42, 43], fraction of modeled information (FMI) and fraction of residual information (FRI) [35], and the Akaike information criterion [7, 43]. These results can be exported to an Excel (office.microsoft.com/en-us/excel) spreadsheet for offline analysis.

#### Estimation of model parameters

All curve fitting functions in ROCKETSHIP are implemented using MATLAB’s Curve Fitting Toolbox. T1, T2 and ADC signal equations can be linearized and fitted with linear regression (See Appendix A). Alternatively, these parameters can be directly fitted with non-linear methods. ROCKETSHIP uses the trust region algorithm provided in the Curve Fitting Toolbox to perform non-linear least squares regression. For T1, T2 and ADC regression, the parameters are hard-coded to have non-negative value constraints. Robust curve fitting is dependent on appropriate starting parameters for the fitting routine [44]. To facilitate this process, a preferences text file defining parameter constraints and convergence criteria, such as fitting tolerances and maximum numerical of iterations, is provided to allow easy editing of these variables. This text file is read by ROCKETSHIP when AIF and model fitting sub-modules are run.

During testing of ROCKETSHIP, it was found that K_trans_ fitting often converged to local minima instead of the desired global minimum solution. To address this, K_trans_ was fitted using multiple starting values with the fit value converging with the lowest residual used as the final value. Other variables were less sensitive to the starting position and thus a single initial value was used to fit each of those variables.

Voxel-wide fitting is performed in parallel using functions provided by MATLAB’s Parallel Computing Toolbox for increased performance.

#### Inputs and outputs

To enable processing of both preclinical and clinical data, the software can read and write both NIFTI/Analyze and DICOM formats. ROIs can be input to ROCKETSHIP as image files drawn in other image visualization programs such as MRIcro (www.mricro.com) or as*. roi* files generated from ImageJ (imagej.nih.gov/ij).

Datasets can be processed through the pipeline individually. A batch mode is also available to generate multiple parametric maps in the *fitting* and DCE-MRI model fitting modules.

Text files logging outputs from each module are stored and emailed to the user upon task completion. Data from each module in the processing pipeline are also stored in files to facilitate debugging, transfer between users and allows processing pauses between modules.

## Results

### Validation of ROCKETSHIP software with simulation

#### Precision and accuracy of individual kinetic model fitting

The robustness of ROCKETSHIP was evaluated with simulations. Simulated datasets containing two (Tofts, Patlak models), three (Extended Tofts) and four parameters (two-compartment exchange model, 2CXM) were generated using MATLAB. The ability of ROCKETSHIP to recover the appropriate DCE-MRI parameter values from simulated data at different SNR and time resolutions was evaluated. To achieve this, curves were generated using specific parameter values defined in Table 2. For each parameter permutation Rician noise at different SNRs was added to the signal intensity versus time curves and fitted. This process was repeated with new noise 100 times in a Monte-Carlo manner to estimate the accuracy and precision of the fits. Resultant curves were fitted with ROCKETSHIP using the same model that was used to generate the curves. The population AIF published by Parker et al. was used in all simulations [15]. Default fitting options were used. The accuracy of the fitted parameters were evaluated with the concordance correlation coefficient (CCC) [45].

**Table 2:** Fitting parameters for simulation studies

*Generating model*	*Fitting model*	*Acquistion duration (min)*	*Time resolution (s)*	*SNR*	*Ktrans (1/min)*	*ve*	*vp*	*Fp (1/min)*	*τi (s)*
*Patlak*	*Patlak*	10	0.5, 6	5, 100	0.01, 0.02, 0.05, 0.1, 0.2, 0.35	N/A	0.001, 0.005 0.01 0.02 0.05 0.1	N/A	N/A
*Tofts*	*Tofts*	10	0.5, 6	5, 100	0.01, 0.02, 0.05, 0.1, 0.2, 0.35	0.01, 0.02, 0.05, 0.1, 0.2, 0.5	N/A	N/A	N/A
*Ex-Tofts*	*Ex-Tofts*	10	0.5, 6	5, 100	0.01, 0.02, 0.05, 0.1, 0.2, 0.35	0.01, 0.02, 0.05, 0.1, 0.2, 0.5	0.001, 0.005 0.01 0.02 0.05 0.1	N/A	N/A
*2CXM*	*2CXM*	10	0.5, 6	5, 100	0.01, 0.02, 0.05, 0.1, 0.2, 0.35	0.01, 0.02, 0.05, 0.1, 0.2, 0.5	0.001, 0.005 0.01 0.02 0.05 0.1	0.5, 1, 5	N/A
*Tissue uptake*	*Tissue uptake*	10	0.5, 6	5, 100	0.01, 0.02, 0.05, 0.1, 0.2, 0.35	N/A	0.001, 0.005 0.01 0.02 0.05 0.1	0.5, 1, 5	N/A
*FXR*	*FXR*	10	0.5, 6	5, 100	0.01, 0.02, 0.05, 0.1, 0.2, 0.35	0.01, 0.02, 0.05, 0.1, 0.2, 0.5	N/A	N/A	0.1, 0.5, 2
*Steady-state*	*Nested*	10	0.5	5, 100	N/A	N/A	0.005, 0.1	N/A	N/A
*Patlak*	*Nested*	10	0.5	5, 100	0.01, 0.35	N/A	0.005, 0.1	N/A	N/A
*Ex-Tofts*	*Nested*	10	0.5	5, 100	0.01, 0.35	0.01, 0.1	0.005, 0.1	N/A	N/A

**Fig. 3:**
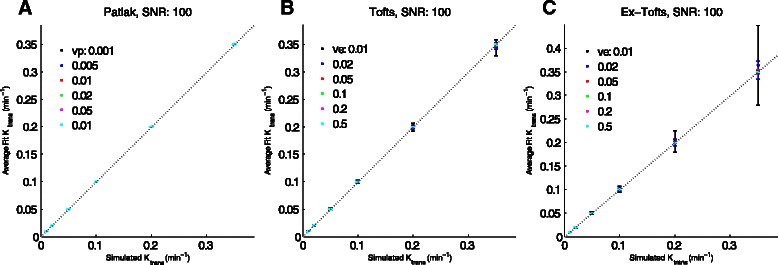
K_trans_ fitting of simulated data. Simulated data with time resolution of 0.5 s and SNR = 100 were fitted using the same model used to generate the simulation with ROCKETSHIP using default settings for the Patlak method (**a**), Tofts (**b**) and Extended Tofts models (**c**). K_trans_ simulated vs. fitted were plotted as a function of v_e_ and v_p_. Dashed line is unity. Error bars denote standard deviation. Given the similar fits, points for different v_e_ and v_p_ may overlap. Concordance correlation coefficients for these (and other model fits) are shown in Tables 3, 4 and 5

**Fig. 4:**
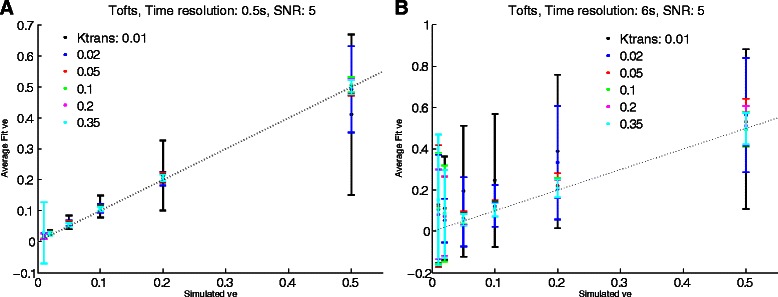
v_e_ fitting at different time resolutions. Simulated data using the Tofts model were generated at SNR = 5 and at time resolutions of 0.5 s (**a**) and 6 s (**b**). Simulated vs. fitted *v*_*e*_ were plotted as a function of K_trans_. Dashed line is unity. Error bars represent standard deviation. As expected, lower time resolution results in a high standard deviation of the curve fits. Given the similar fits, points for different K_trans_ may overlap. Concordance correlation coefficients for these (and other model fits) are shown in Tables 3, 4 and 5

Figure 3 shows plots comparing fitted K_trans_ values to the values used to generate the simulated curves for different models. At high SNR and short time resolution, there is good concordance between simulated and fitted values. This is reflected in the CCC comparisons (Tables 3, 4 and 5). Fitting using ROCKETSHIP was generally able to recover parameter values with high accuracy and precision, as demonstrated by the small error bars seen in Fig. 3 and the high CCC values for the fitted to actual value comparisons across multiple models and with different parameters. Results shown in Fig. 3 and Tables 3, 4 and 5 demonstrate that ROCKETSHIP perform on par or better compared to QIBA simulation fittings using the Tofts model with DCE@UrLAB and DCEMRI.jl both qualitatively (compared to figures generated by DCE@UrLAB [33]) and quantitatively (CCC ≥ 0.9 in general for K_trans_ and v_e_ recovery using Tofts model, similar to or better than the reported CCCs generated by DCEMRI.jl [28]). As with all curve fitting algorithms, the accuracy of the fit will be dependent on a number of factors [18], including SNR, sampling resolution and the number of parameters being fitted (Fig. 4, Tables 3, 4 and 5).

**Table 3:** Concordance correlation coefficients (CCC) comparing fitted and simulated Ktrans using different models and dependent parameters

Generating model	Fitting model	Time resolution (s)	SNR	Dependent parameter	*vp = 0.001*	*0.005*	*0.01*	*0.02*	*0.05*	*0.1*
*Patlak*	*Patlak*	0.5	5	*vp*	1.00	1.00	1.00	1.00	1.00	1.00
		0.5	100	*vp*	1.00	1.00	1.00	1.00	1.00	1.00
		6	5	*vp*	0.98	0.98	0.98	0.98	0.98	0.98
		6	100	*vp*	1.00	1.00	1.00	1.00	1.00	1.00
					*ve = 0.01*	*0.02*	*0.05*	*0.1*	*0.2*	*0.5*
*Tofts*	*Tofts*	0.5	5	*ve*	0.38	0.85	0.98	0.99	1.00	1.00
		0.5	100	*ve*	1.00	1.00	1.00	1.00	1.00	1.00
		6	5	*ve*	0.08	0.32	0.76	0.92	0.96	0.97
		6	100	*ve*	0.67	0.92	1.00	1.00	1.00	1.00
					*ve = 0.01*	*0.02*	*0.05*	*0.1*	*0.2*	*0.5*
*Ex-Tofts*	*Ex-Tofts*	0.5	5	*ve*	0.01	0.33	0.95	0.99	1.00	1.00
		0.5	100	*ve*	0.92	1.00	1.00	1.00	1.00	1.00
		6	5	*ve*	−0.02	0.05	0.41	0.84	0.96	0.98
		6	100	*ve*	0.22	0.48	0.98	1.00	1.00	1.00
					*vp = 0.001*	*0.005*	*0.01*	*0.02*	*0.05*	*0.1*
		0.5	5	*vp*	0.74	0.73	0.71	0.68	0.61	0.51
		0.5	100	*vp*	0.99	0.98	0.98	0.99	0.99	0.98
		6	5	*vp*	0.57	0.58	0.57	0.56	0.45	0.41
		6	100	*vp*	0.89	0.90	0.88	0.81	0.54	0.35
					*ve = 0.01*	*0.02*	*0.05*	*0.1*	*0.2*	*0.5*
*2CXM*	*2CXM*	0.5	5	*ve*	−0.02	−0.02	0.11	0.22	0.65	0.90
		0.5	100	*ve*	0.07	0.42	0.65	0.76	0.98	0.99
		6	5	*ve*	−0.01	−0.01	−0.01	0.10	0.52	0.84
		6	100	*ve*	−0.03	−0.06	0.04	0.38	0.84	0.98
					*vp = 0.001*	*0.005*	*0.01*	*0.02*	*0.05*	*0.1*
		0.5	5	*vp*	0.18	0.20	0.23	0.31	0.43	0.47
		0.5	100	*vp*	0.32	0.63	0.72	0.76	0.76	0.74
		6	5	*vp*	0.20	0.19	0.21	0.21	0.28	0.29
		6	100	*vp*	0.18	0.26	0.34	0.41	0.45	0.45
					*Fp = 0.5*	*1*	*5*			
		0.5	5	*Fp*	0.21	0.30	0.40			
		0.5	100	*Fp*	0.46	0.69	0.81			
		6	5	*Fp*	0.20	0.22	0.27			
		6	100	*Fp*	0.26	0.35	0.43			
					*vp = 0.001*	*0.005*	*0.01*	*0.02*	*0.05*	*0.1*
					*vp = 0.001*	*0.005*	*0.01*	*0.02*	*0.05*	*0.1*
		0.5	5	*vp*	0.98	1.00	0.98	1.00	1.00	1.00
		0.5	100	*vp*	1.00	1.00	1.00	1.00	1.00	1.00
		6	5	*vp*	0.88	0.88	0.88	0.91	0.98	0.99
		6	100	*vp*	1.00	1.00	1.00	1.00	1.00	1.00
*Tissue uptake*	*Tissue uptake*	0.5	5	*vp*	0.98	1.00	0.98	1.00	1.00	1.00
		0.5	100	*vp*	1.00	1.00	1.00	1.00	1.00	1.00
		6	5	*vp*	0.88	0.88	0.88	0.91	0.98	0.99
		6	100	*vp*	1.00	1.00	1.00	1.00	1.00	1.00
					*Fp = 0.5*	*1*	*5*			
		0.5	5	*Fp*	1.00	0.98	1.00			
		0.5	100	*Fp*	1.00	1.00	1.00			
		6	5	*Fp*	0.82	0.97	0.98			
		6	100	*Fp*	1.00	1.00				
					*ve = 0.01*	*0.02*	*0.05*	*0.1*	*0.2*	*0.5*
					*ve = 0.01*	*0.02*	*0.05*	*0.1*	*0.2*	*0.5*
		0.5	5	*ve*	−0.01	0.02	0.08	0.14	0.39	0.95
		0.5	100	*ve*	0.07	0.31	0.97	1.00	1.00	1.00
		6	5	*ve*	0.01	−0.01	0.03	0.06	0.12	0.38
		6	100	*ve*	0.05	0.14	0.71	0.96	0.99	1.00
					*τi = 0.1*	*0.5*	*2*			
		0.5	5	*τi*	0.11	0.14	0.14			
		0.5	100	*τi*	0.56	0.59	0.56			
		6	5	*τi*	0.06	0.07	0.07			
		6	100	*τi*	0.54	0.61	0.52			

**Fig. 5:**
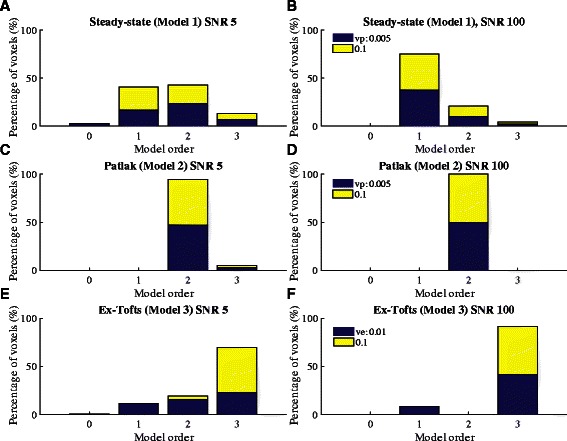
Nested model selection from simulated data. **a** and **b** show fitting for steady-state model simulated data. **c** and **d** show the fitting for Patlak simulated data. All the generated curves at SNR = 100 converged to the correct model. At lower SNR, some of the curves incorrectly converged to Model 3 (extended Tofts). **e** and **f** show fitting on extended Tofts simulated data. Again, the majority of the curves converged to the correct model. The percentage of voxels attributed to each model by the nest model algorithm is shown in Table 6

100 curves for each model and fixed dependent parameter were generated as described in the text and Table 2. Ktrans values simulated are defined in Table 2. The CCC was calculated from the Ktrans (simulated) vs. Ktrans (fitted), such as depicted in Figure 5. Ktrans values from which CCCs were calculated were segregated according to the dependent parameter (vp, ve or Fp*).* A value of 1 shows near-perfect concordance, while 0 represents a low concordance relationship

**Table 4:** Concordance correlation coefficients (CCC) comparing fitted and simulated vp using different models and dependent parameters

Generating model	Fitting model	Time resolution (s)	SNR	Dependent parameter	*Ktrans = 0.01*	*0.02*	*0.05*	*0.1*	*0.2*	*0.35*
*Patlak*	*Patlak*	0.5	5	*Ktrans*	0.99	0.99	0.99	0.99	1.00	0.99
		0.5	100	*Ktrans*	1.00	1.00	1.00	1.00	1.00	1.00
		6	5	*Ktrans*	0.95	0.95	0.95	0.95	0.94	0.95
		6	100	*Ktrans*	1.00	1.00	1.00	1.00	1.00	1.00
					*Ktrans = 0.01*	*0.02*	*0.05*	*0.1*	*0.2*	*0.35*
*Ex-Tofts*	*Ex-Tofts*	0.5	5	*Ktrans*	0.99	0.99	0.99	0.99	0.98	0.98
		0.5	100	*Ktrans*	1.00	1.00	1.00	1.00	1.00	1.00
		6	5	*Ktrans*	0.95	0.95	0.94	0.93	0.91	0.88
		6	100	*Ktrans*	1.00	1.00	1.00	1.00	0.95	0.89
					*ve = 0.01*	*0.02*	*0.05*	*0.1*	*0.2*	*0.5*
		0.5	5	*ve*	0.98	0.98	0.99	0.99	0.99	0.99
		0.5	100	*ve*	1.00	1.00	1.00	1.00	1.00	1.00
		6	5	*ve*	0.92	0.90	0.89	0.93	0.95	0.94
		6	100	*ve*	0.92	0.92	1.00	1.00	1.00	1.00
					*Ktrans = 0.01*	*0.02*	*0.05*	*0.1*	*0.2*	*0.35*
*2CXM*	*2CXM*	0.5	5	*Ktrans*	0.79	0.85	0.51	0.26	0.10	0.06
		0.5	100	*Ktrans*	1.00	1.00	0.95	0.96	0.81	0.41
		6	5	*Ktrans*	0.02	0.07	0.37	0.16	0.07	0.05
		6	100	*Ktrans*	0.96	0.93	0.90	0.73	0.38	0.09
					*ve = 0.01*	*0.02*	*0.05*	*0.1*	*0.2*	*0.5*
		0.5	5	*ve*	0.95	0.90	0.65	0.31	0.15	0.02
		0.5	100	*ve*	0.99	0.97	0.89	0.75	0.82	0.57
		6	5	*ve*	0.44	0.56	0.35	0.11	0.02	−0.03
		6	100	*ve*	0.97	0.93	0.75	0.51	0.34	0.13
					*Fp = 0.5*	*1*	*5*			
		0.5	5	*Fp*	0.22	0.28	0.31			
		0.5	100	*Fp*	0.64	0.88	0.95			
		6	5	*Fp*	0.09	0.09	0.03			
		6	100	*Fp*	0.39	0.56	0.54			
					*Ktrans = 0.01*	*0.02*	*0.05*	*0.1*	*0.2*	*0.35*
*Tissue uptake*	*Tissue uptake*	0.5	5	*Ktrans*	0.95	0.78	0.18	0.03	−0.02	−0.03
		0.5	100	*Ktrans*	1.00	1.00	1.00	1.00	1.00	0.48
		6	5	*Ktrans*	0.33	0.39	0.31	0.17	0.02	0.01
		6	100	*Ktrans*	0.74	0.83	0.79	0.80	0.77	0.69
					*Fp = 0.5*	*1*	*5*			
		0.5	5	*Fp*	-0.01	-0.01	0.90			
		0.5	100	*Fp*	0.67	1.00	0.99			
		6	5	*Fp*	0.03	0.20	0.07			
		6	100	*Fp*	0.95	0.97	0.44			

100 curves for each model and fixed dependent parameter were generated as described in the text and Table 2. vp values simulated are defined in Table 2. The CCC was calculated from the vp (simulated) vs. vp (fitted) . vp values from which CCCs were calculated were segregated according to the dependent parameter (Ktrans, ve or Fp*).* A value of 1 shows near-perfect concordance, while 0 represents a low concordance relationship

**Table 5:** Concordance correlation coefficients (CCC) comparing fitted and simulated ve using different models and dependent parameters

						CCC for ve (simulated) vs. ve (fitted)				
Generating model	Fitting model	Time resolution (s)	SNR	Dependent parameter	*Ktrans = 0.01*	*0.02*	*0.05*	*0.1*	*0.2*	*0.35*
*Tofts*	*Tofts*	0.5	5	*Ktrans*	0.71	0.96	1.00	1.00	0.94	0.97
		0.5	100	*Ktrans*	1.00	1.00	1.00	1.00	1.00	1.00
		6	5	*Ktrans*	0.33	0.52	0.68	0.63	0.65	0.68
		6	100	*Ktrans*	0.95	1.00	1.00	1.00	1.00	1.00
					*Ktrans = 0.01*	*0.02*	*0.05*	*0.1*	*0.2*	*0.35*
*Ex-Tofts*	*Ex-Tofts*	0.5	5	*Ktrans*	0.67	0.86	0.87	0.80	0.66	0.54
		0.5	100	*Ktrans*	1.00	1.00	1.00	1.00	1.00	1.00
		6	5	*Ktrans*	0.22	0.40	0.48	0.47	0.35	0.35
		6	100	*Ktrans*	0.95	1.00	1.00	1.00	0.93	0.74
					*vp = 0.001*	*0.02*	*0.05*	*0.1*	*0.2*	*0.5*
		0.5	5	*vp*	0.74	0.73	0.69	0.71	0.73	0.70
		0.5	100	*vp*	1.00	1.00	1.00	1.00	1.00	1.00
		6	5	*vp*	0.38	0.38	0.36	0.36	0.32	0.32
		6	100	*vp*	0.92	0.94	0.93	0.93	0.93	0.94
					*Ktrans = 0.01*	*0.02*	*0.05*	*0.1*	*0.2*	*0.35*
*2CXM*	*2CXM*	0.5	5	*Ktrans*	0.51	0.52	0.29	0.19	0.12	0.06
		0.5	100	*Ktrans*	0.97	0.97	0.93	0.84	0.70	0.49
		6	5	*Ktrans*	0.17	0.27	0.24	0.15	0.06	0.03
		6	100	*Ktrans*	0.81	0.88	0.73	0.46	0.23	0.10
					*vp = 0.001*	*0.02*	*0.05*	*0.1*	*0.2*	*0.5*
		0.5	5	*vp*	0.09	0.15	0.24	0.36	0.41	0.37
		0.5	100	*vp*	0.50	0.83	0.88	0.91	0.87	0.86
		6	5	*vp*	0.11	0.12	0.14	0.16	0.21	0.19
		6	100	*vp*	0.23	0.41	0.49	0.55	0.59	0.63
					*Fp = 0.5*	*1*	*5*			
		0.5	5	*Fp*	0.19	0.24	0.31			
		0.5	100	*Fp*	0.68	0.81	0.90			
		6	5	*Fp*	0.14	0.15	0.17			
		6	100	*Fp*	0.40	0.48	0.54			
*FXR*	*FXR*	0.5	5	*Ktrans*	0.37	0.41	0.43	0.48	0.52	0.46
		0.5	100	*Ktrans*	0.90	0.92	0.96	0.93	0.93	0.94
		6	5	*Ktrans*	0.20	0.23	0.25	0.24	0.23	0.23
		6	100	*Ktrans*	0.63	0.76	0.80	0.81	0.74	0.82
					*τi = 0.1*	*0.5*	*2*			
		0.5	5	*τi*	0.41	0.45	0.44			
		0.5	100	*τi*	0.94	0.93	0.92			
		6	5	*τi*	0.24	0.23	0.22			
		6	100	*τi*	0.76	0.75	0.73			

100 curves for each model and fixed dependent parameter were generated as described in the text and Table 2. ve values simulated are defined in Table 2. The CCC was calculated from the ve (simulated) vs. ve (fitted) . ve values from which CCCs were calculated were segregated according to the dependent parameter (Ktrans, vp or Fp*).* A value of 1 shows near-perfect concordance, while 0 represents a low concordance relationship

#### Accuracy of model selection using nested model analysis

The data-driven, nested model analysis function in ROCKETSHIP was also tested. Simulation curves reflective of each level of nesting (steady-state, Patlak and extended Tofts models) were generated as above. The resultant curves were fitted using the nested method.

Simulation results using the nested model fitting are shown in Fig. 5 and Table 6. The nested model fitting was able to recover the appropriate model for the majority of the fitting curves. Accuracy of the nested model fitting was shown to be dependent on SNR. Lower SNR led to more model misclassification of voxels.

**Table 6:** Model selection of simulated data using the nested model method

		*Percentage of voxels selected (%)*			
Generating model	SNR	Model 0	Model 1	Model 2	Model 3
*Steady-State (Model 1)*	5	2.75	41	43	13.25
*Steady-State (Model 1)*	100	0	75	20.75	4.25
*Patlak (Model 2)*	5	0	0	94.75	5.25
*Patlak (Model 2)*	100	0	0	100	0
*Extended Tofts (Model 3)*	5	0	11.25	19	69.5
*Extended Tofts (Model 3)*	100	0	8.5	0	91.5

### In vivo examples

#### Preclinical data set

DCE-MRI was performed on an athymic BLABL/c nude mouse bearing a HER2-expressing BT474 human breast cancer tumor. A 17^β^-estradiol pellet (Innovative Research of America) was implanted subcutaneously into the back of the mouse one day prior to orthotopic inoculation (6^th^ and 9^th^ mammary fat pad) of 5 × 10^6^ cells in matrigel (BD Biosciences). The tumor volume was ≈ 200 mm^3^ at the time of imaging. Mouse care and experimental procedures were carried out in accordance with protocols approved by the Institutional Animal Care and Use Committee at Caltech.

Imaging was performed using a Biospec (Bruker-Biospin Inc. Billerica, MA) 7 T MRI scanner and a custom-built birdcage coil. The mouse was anesthetized during the session with 1.3-1.5 % isoflurane/air mixture. Body temperature was maintained at 36-37 °C with warmed air through the bore. A variable flip angle method was used to generate T1 maps. Gradient echo images (FLASH, FA = 12°, 24°, 36°, 48°, 60°, matrix size = 140 × 80, voxel size = 0.25 × 0.25 mm^2^, slice thickness = 1 mm, TR/TE = 200/2 ms) centered on the tumor (3 slices) and the left ventricle (1 slice) were acquired. Next, DCE-MRI was acquired (FLASH, FA = 35°, TR/TE = 25/2 ms, geometry the same as the T1 maps, time resolution = 2 s, duration = 22 min). After a baseline of 2.5 min, Gd-DTPA (0.1 mmol/kg, Magnevist, Bayer) was injected intravenously via a tail vein catheter using a powered-injector (New Era Inc.) at 0.5 mL/min. Imaging data were processed using ROCKETSHIP and analyzed with the nested model method.

**Fig. 6:**
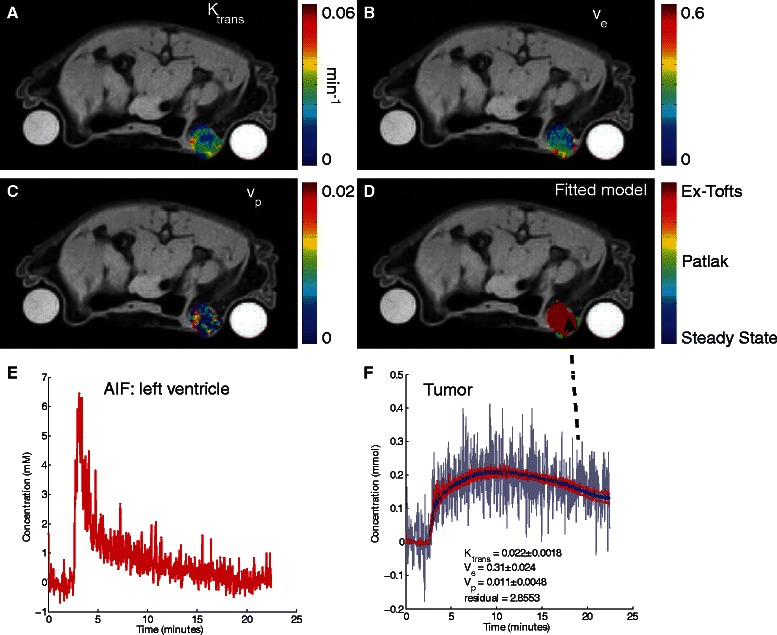
Nested model fitting of DCE-MRI data on a murine breast cancer tumor model. Parameters for K_trans_ (**a**), v_e_ (**b**), and v_p_ (**c**) are shown. As shown in **d**, the majority of the voxels fitted best to the extended Tofts model, with some edge voxels fitting to the Patlak method. **e** shows the AIF used for the fit (taken from the left ventricle). **f** shows a sample time curve from the edge of the tumor (denoted by arrow) with corresponding fit (blue denotes the fit, red lines denote the 95 % prediction bounds for the fitted curve). Rod phantoms on either side of the mouse were present to allow for signal drift correction (not used in this case)

Parametric maps and associated concentration vs. time curves are shown in Fig. 6. For this tumor, the extended Tofts model was deemed the most appropriate for the majority of the tumor. Generated K_trans_ values were similar to those observed in prior studies using the same tumor cell line [46]. Consistent with other studies, there is a heterogeneous distribution of K_trans_, v_e_ and v_p_ highlighting a leaky and vascularized rim with a necrotic core.

#### Clinical data set

The clinical data set was obtained from a wider study set being accrued by the University of Southern California (USC) Alzheimer’s disease Research Center. The study was approved by the USC Institutional Review Board. The imaging dataset used here was obtained from one of the participants from the no cognitive impairment cohort, as determined from medical examination and neuropsychological evaluation. All imaging was performed at the Keck Medical Center of USC. Participants underwent a medical examination, neuropsychological evaluations and blood draw to ensure appropriate kidney function for CA administration prior to imaging.

The imaging protocol performed was developed to study BBB changes in patients with cognitive impairment and is detailed in [6]. Briefly, all images were obtained on a GE 3 T HDXT MR scanner with a standard eight-channel array head coil. Anatomical coronal spin echo T2-weighted scans were first obtained through the hippocampi (TR/TE 1550/97.15 ms, NEX = 1, slice thickness 5 mm with no gap, FOV = 188 x 180 mm, matrix size = 384 x 384). Baseline coronal T1-weighted maps were then acquired using a T1-weighted 3D spoiled gradient echo (SPGR) pulse sequence and variable flip angle method using flip angles of 2°, 5° and 10°. (TR/TE = 8.29/3.08 ms, NEX = 1, slice thickness 5 mm with no gap, FOV 188 x 180 mm, matrix size 160 x 160). Coronal DCE MRI covering the hippocampi and temporal lobes were acquired using a T1-weighted 3D SPGR pulse sequence (FA = 15°, TR/TE = 8.29/3.09 ms, NEX = 1, slice thickness 5 mm with no gap, FOV 188 x 180 mm, matrix size 160 x 160, voxel size was 0.625 x 0.625 x 5 mm^3^). This sequence was repeated for a total of 16 min with an approximate time resolution of 15.4 s. Gadobenate dimegulumine (MultiHance®, Braaco, Milan, Italy) (0.05 mmol/kg), a gadolinium-based CA, was administered intravenously into the antecubital vein using a power injector, at a rate of 3 mL/s followed by a 25 mL saline flush, 30 s into the DCE scan. Imaging data were processed by ROCKETSHIP. The AIF, which was extracted from an ROI positioned at the internal carotid artery, was fitted with a bi-exponential function prior to fitting analysis with 2CXM.

**Fig. 7:**
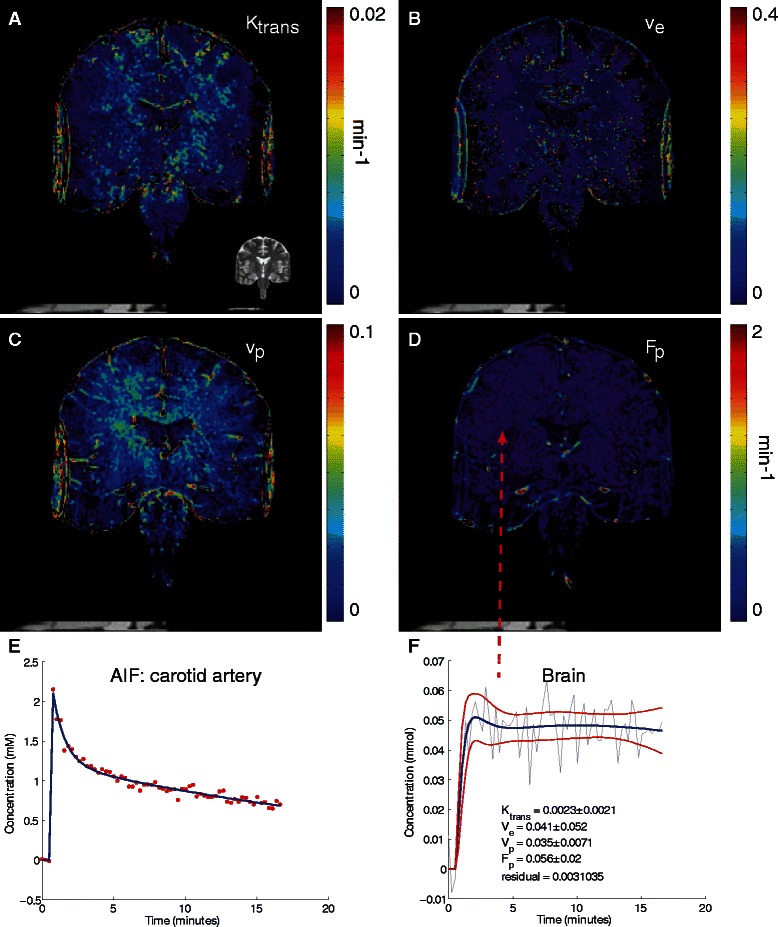
2CXM fitting of a normal human brain. Parameters for K_trans_ (**a**), v_e_ (**b**), v_p_ (**c**) and F_p_ (**d**) are shown. **e** shows the AIF used (taken from internal carotid artery). The AIF was fitted with a bi-exponential curve (blue) prior to tissue fitting. **f** shows a sample time curve from the brain parenchyma (denoted by arrow) with corresponding fit (blue denotes the fit, red lines denote the 95 % prediction bounds for the fitted curve). Fold over artifact is seen on the lateral brain edges due to truncation by the field of view bounding box

Parametric maps and associated concentration vs. time curves are shown in Fig. 7. Compared to the murine tumor that contains leaky vasculature [41], K_trans_ values for the human brain are lower, as expected given the intact blood brain barrier in normal human subjects [6]. As shown in Fig. 7e, the bi-exponential function estimated the AIF well and allowed for a good fit of concentration time curves within the brain parenchyma (Fig. 7f).

## Discussion

Development and adoption of DCE-MRI by clinicians and researchers requires the availability of analysis software that not only has sufficient functionality for the novice end-user, but also the flexibility and extensibility capability for more complicated analyses. Using its default settings, users can follow ROCKETSHIP’s pipeline to generate parametric maps of common kinetic models. Its modular design allows other models to be incorporated in a straightforward manner. Recently, several groups have recognized that data-driven approaches in selecting the appropriate model may be important for proper physiological interpretation of the DCE-MRI data [23]. To facilitate this, ROCKETSHIP includes a nested model fitting option as well as statistical tools to analyze and compare the goodness-of-fit between different kinetic models.

We selected the MATLAB environment for a number of reasons. Firstly, MATLAB is widely used for image analysis in the academic community; programs written in MATLAB can generally be run on Mac, Windows or Unix-based operating systems with minimal porting issues. Secondly, MATLAB provides several toolboxes (such as the Curve Fitting Toolbox and Parallel Computing Toolbox) that facilitated development of the current software and allows future extensions to be easily implemented. MATLAB also has a strong user-contributed library of imaging processing and statistical analysis functions that aid this endeavor. Building and testing of the software are helped by the interpretive nature of the MATLAB language. Modifications to the existing code or testing of new modules can be performed in real time. These capabilities enable users of wide-ranging expertise (from experienced programmers to biologists/clinicians with less programming experience) to use ROCKETSHIP and develop new modules and extend the software.

Since it is run within the MATLAB environment, the execution time of ROCKETSHIP may be slower than other DCE-MRI software written in compiled languages (C, C++, IDL). The use of the Parallel Computing Toolboxto do the voxel-by-voxel processing in parallel mostly offsets this limitation. While this may be a disadvantage in a daily clinical workflow setup, most DCE-MRI data are analyzed offline and often in a research context. As discussed above, ROCKETSHIP will be advantageous in this scenario. Furthermore, MATLAB code can be ported to C/C++ and Julia [28] in a relatively straightforward way. Thus, once an optimal setup is developed using ROCKETSHIP, one can translate this to a lower level language and ultimately as a standalone program.

The precision and accuracy of DCE-MRI parameter estimation in vivo is dependent on several factors, ranging from the type of tissue being probed, the type of image acquisition protocol/system used to the post-acquisition processing and analysis methodologies. DCE-MRI software packages address the post-acquisition portion of this pipeline. To evaluate ROCKETSHIP’s ability in this regard, simulation datasets with a range of models and parameters expected in typical studies were generated and fitted. Results demonstrate that ROCKETSHIP was able to recover DCE-MRI parameters accurately using its default configuration, on par with (or better than) similar studies using the same pharmacokinetic models implemented within currently available software packages. Furthermore, the nested model functionality, unavailable in prior software suites, was generally able to select the appropriate kinetic model for a given simulation dataset. ROCKETSHIP was also able to fit both preclinical and clinical in vivo DCE-MRI data well, demonstrating its applicability for in vivo studies.

While the simulations presented cover a broad parameter range, it is likely that further modifications/tuning of ROCKETSHIP’s settings or functions will be required for specific in vivo applications in the future. Furthermore, extrinsic factors affecting the data fitting, including SNR, time resolution/duration, motion and most importantly the physiological question being explored, need to be considered when evaluating the appropriateness of the output values and model selection [23, 37, 47, 48].

## Conclusion

We have implemented a modular, flexible and easily extensible software suite for dynamic MRI (in particular DCE-MRI) analysis. This software allows for DCE-MRI data analysis using several pharmacokinetic models used currently in the literature as well as data-driven analysis methods. It is compatible with both clinical and preclinical imaging data and is currently being used in DCE-MRI studies [6]. We envision that the flexibility and open source nature of our software will be useful for researchers and clinicians at varying levels of DCE-MRI expertise.

The source code repository and documentation for ROCKETSHIP is located at https://github.com/petmri/ROCKETSHIP. Future updates and support for ROCKETSHIP will be coordinated and maintained via this repository.

## Availability and requirements

Project name: ROCKETSHIP v.1.1

Project homepage: https://github.com/petmri/ROCKETSHIP

Operating system(s): Windows/Mac OS X/Linux

Programming language: MATLAB

Other requirements: None, but image processing programs such as ImageJ or MRIcro are useful to pre-process inputs.

License: GPL-2.0

## Appendix A: MRI image parameter fitting

### T2/T2* and ADC fitting

The current implementation of the *fitting* module provides options to fit T1, T2/T2* and ADC maps. T2/T2* fits are based on the mono-exponential equation, while ADC is based on the mono-exponential Steksjal-Tanner equation [49]. Options to perform a non-linear least squares fit or a linear regression using a linearized form of the equations are provided.

### T1 fitting

T1 relaxation times estimation in ROCKETSHIP can be performed using the variable TR method, the variable flip-angle (FA) method and the inversion recovery (IR) method.

Using the variable TR method, T1 is estimated with a series of spin-echo images with varying TR and constant TE using the standard saturation recovery equation:

A1 $$ SI=S{I}_{TR=\infty\ }\left(1-{e}^{-\frac{TR}{T1}}\right).\kern0.5em {e}^{-TE/T2} $$

The variable FA method uses a series of gradient-echo images with varying FA and fixed TR and TE to estimate T1 with the equation:

A2 $$ SI=S{I}_{\left(TR\gg T{1}_{,\kern0.5em }TE\ll T{2}^{*},\kern0.5em \theta ={90}^{\circ}\right)}\frac{ \sin \theta \left(1-{E}_1\right){E}_2}{1-{E}_1 \cos \theta } $$

where *θ* is the FA, ${E}_1 = {e}^{-\frac{TR}{T1}}$ and ${E}_2 = {e}^{-TE/T{2}^{*}}$. Here, we assume that TE < T2*.

The “gold-standard” IR method of T1 estimation consists of inverting the longitudinal magnetization and sampling the MR signal at several points along its exponential recovery. An IR pulse sequence is repeated *N* times with an inversion pulse followed by an imaging module delayed by different waiting times (TI_N_). T1 can be estimated with the equation:

A3 $$ SI = S{I}_{\left(TR,\ TI\gg T1\right)}\left(1-2.{e}^{-\frac{TI}{T1}} + {e}^{-\frac{TR}{T1}}\right) $$

Non-linear least-squares fitting is used to fit MRI data to Equations A1, A2, A3.

## Appendix B: Preparation of the dynamic datasets for DCE-MRI analysis

### Signal to concentration conversion

ROCKETSHIP assumes that dynamic DCE-MRI data are acquired with a spoiled-gradient echo sequence. Files can be read in as 2D or 3D image sequences or as a 4D image volume. SI from the raw data images are converted to R_1_(t) curves using the equation:

B1 $$ {R}_1(t)=\left(-\frac{1}{TR}\right). \log \frac{\left[{S}_0 \sin \theta\ {e}^{-\frac{TE}{T{2}^{*}}}-S(t) \cos \theta \right]}{\left[{S}_0 \sin \theta\ {e}^{-\frac{TE}{T{2}^{*}}}-S(t)\right]} $$

where θ is the FA, S_0_ represents the fully relaxed situation and is calculated by:

B2 $$ {S}_0=\frac{S_{ss}\left[1-{e}^{-\frac{TR}{T1}} \cos \theta \right]}{\left[1-{e}^{-\frac{TR}{T1}}\right] \sin \theta .{e}^{-\frac{TE}{T{2}^{*}}}} $$

S_ss_ is the average steady state intensity prior to contrast agent injection.

Assuming fast exchange limit [40], resultant R_1_(t) curves are converted to concentration curves, C(t), via the relations:

B3 $$ {C}_{AIF}(t) = \left({R}_1(t)-\frac{1}{T1}\right)/\Big({r}_1.\left(1-HCT\right) $$

, and

B4 $$ {C}_{tissue}(t) = \left({R}_1(t)-\frac{1}{T1}\right)/{r}_1 $$

where r_1_ is the relaxivity of the contrast agent and HCT hematocrit of blood.

### Noise filtering

Voxels where Equation B1 yields imaginary values are removed from the ROIs being evaluated. Further voxels are filtered out if they are too noisy. The signal-to-noise ratio (SNR) threshold and the region from which the noise is calculated are defined by the user.

### Signal drift correction

Baseline signal intensity can drift during the course of a dynamic study. If the user has a phantom in the field-of-view, ROCKETSHIP can use the signal from this phantom to correct the dynamic images for drift. In the current implementation, the signal drift in the phantom over time is used to generate a multiplicative scaling factor by taking the ratio of the phantom image intensity at time t compared to the initial time point. To minimize the effects of aberrant noise on factor estimation, scaling factors over the time series are fitted to a fourth order polynomial, with the factors derived from this curve used for drift correction.

### AIF extraction

Accurate delineation of the AIF/RR ROI is important for all the quantitative models currently used in ROCKETSHIP. Users manually define the AIF/RR ROI by inputting an image or ImageJ .roi file showing the location of the AIF/RR. SNR filtering is applied as above. For AIF’s, additional options are available to accept only those voxels that demonstrate expected AIF behavior (steady baseline, with a sudden sharp rise in the signal during injection followed with rapid washout). This is achieved using a combination of moving average, Sobel edge-emphasizing filters and fitting to a bi-exponential curve. Thresholds for these filters are defined in the user-editable preference text file.

Data output from this sub-module is saved as a MATLAB .mat file that can be passed down the pipeline for further processing.

## Appendix C: AIF/RR processing

ROCKETSHIP processes AIF/RR curves in three ways, depending on the application:

1. The raw AIF/RR time curves are used for model fitting.
2. The raw AIF curve is fitted to a bi-exponential with a linear upslope during injection of the contrast agent [50]. The equation used to fit the curve is:
  C1 $$ \begin{array}{l}{C}_{AIF}(t)={C}_{ss}, \kern0.5em for\ t < {t}_{i1}\\ {}{C}_{AIF}(t) = {C}_{ss}+\left(A-{C}_{ss}\right).\frac{t-{t}_{i1}}{t_{i2}-{t}_{i2}}-\left(B-{C}_{ss}\right).\frac{t-{t}_{i1}}{t_{i2}-{t}_{i2}},\kern0.5em for\ {t}_{i1}\le t\le {t}_{i2}\\ {}{C}_{AIF}(t)=A.{e}^{-c.\left(t-{t}_{i2}\right)}+B.{e}^{-d.\left(t-{t}_{i2}\right)},\kern0.5em for\ t < {t}_{i2}\end{array} $$
  where C_ss_ is the concentration of contrast agent in the tissue prior to injection (nominally = 0), t_i1_ and t_i2_ the time of onset and end for contrast agent injection respectively. A, B, c and d are variables that are estimated using a non-linear least-squares fit.

3) The AIF/RR curve to be used downstream in the pipeline can be imported from an external file.

A tool is provided for the user to create a population-averaged AIF/RR as well.

Time resolution and duration during acquisition can significantly affect kinetic modeling results [48]. Options to define the time resolution of each sample point and the duration interval to be considered downstream in the pipeline are thus available.

## Appendix D: Pharmacokinetic model fitting

ROCKETSHIP implements several common pharmacokinetic models used for DCE-MRI analysis as well as semi-quantitative metrics. A brief description of these models is presented. The GUI for this sub-module is shown in Fig. 3d.

### Area under curve (AUC)

AUC is calculated using both C(t) and the raw signal curve S(t). AUC is estimated using the trapezoidal rule function implemented in MATLAB. Four parameters are generated: AUC_C(t)_, AUC_S(t)_, NAUC_C(t)_ and NAUC_S(t)_. NAUC the normalized AUC, is defined by:

D1 $$ NAUC = AU{C}_{ROI}/ AU{C}_{AIF/RR} $$

where AUC_ROI_ is the AUC of the tissue curve and AUC_AIF/RR_ is the AUC of the AIF/RR.

### Two-compartment exchange model (2CXM)

Most of the models implemented in ROCKETSHIP are based on the 2CXM [12]. ROCKETSHIP implements the 2CXM using the convolution equation for C_tissue_(t):

D2 $$ {C}_{tissue}(t) = {F}_p.K \otimes {C}_{AIF}(t) $$

where,

D3 $$ K(t) = {e}^{-t.{K}_{+}}+{E}_{-}\left({e}^{-t.{K}_{-}} - {e}^{-{K}_{+}}\right) $$

D4 $$ {K}_{\pm }=\frac{1}{2}\left({T}_P^{-1}+{T}_E^{-1}\pm \sqrt{{\left({T}_P^{-1}+{T}_E^{-1}\right)}^2-4.{T}_E^{-1}.{T}_B^{-1}}\right) $$

D5 $$ {E}_{-} = \frac{K_{+}-{T}_B^{-1}}{K_{+}-{K}_{-}} $$

D6 $$ {T}_P=\frac{v_P}{PS+{F}_P},\ {T}_E = \frac{v_E}{PS},\ {T}_B = \frac{v_p}{F_P} $$

where C_tissue_(t) and C_AIF_(t) are the contrast agent concentration versus time curves for the tissue of interest (imaging voxel) and AIF respectively, v_p_ and v_e_ are the plasma and interstitial volume fractions within the imaging voxel respectively, F_p_ is the flow of plasma carrying the contrast agent into and out of the voxel, PS is the permeability-surface product exchange constant governing transfer of contrast agent between the plasma and interstitial space and ⊗ denotes convolution.

For most studies, an important physiological parameter of interest is K_trans_, the volume transfer constant describing the number of contrast agent molecules delivered to the interstitial space per unit time, tissue volume and arterial plasma concentration. For compartmental models, it was shown previously [12] that:

D7 $$ {K}_{trans}=\frac{F_p.\ PS}{F_p+PS} $$

Thus, four parameters are estimated for the 2CXM: F_p_, v_p_, v_e_, and K_trans_

### Tissue uptake model

This model assumes that there is no backflux of contrast agent from the interstitium back to the plasma compartment, which may apply soon after bolus injection. In this case, v_e_ is not measurable. Three parameters, v_p_, F_p_ and K_trans_, can be estimated using:

D8 $$ {C}_{tissue}(t)={C}_{AIF} \otimes g(t) $$

where

D9 $$ g(t) = {F}_p{e}^{-\frac{t}{T_P}} + {K}_{trans}\left(1-{e}^{-\frac{t}{T_P}}\right) $$

### Tofts and extended Tofts models

The extended Tofts model assumes that F_p_ ≈ ∞ [12]. Three parameters, v_p_, v_e_ and K_trans_, are estimated using:

D10 $$ {C}_{tissue}(t) = {K}_{trans}.{C}_{AIF} \otimes f(t) + {v}_p.{C}_{AIF}(t) $$

where

D11 $$ f(t) = {e}^{-\frac{K_{trans}.t}{v_e}} $$

The Tofts model assumes that the plasma compartment has negligible volume (v_p_ = 0):

D12 $$ {C}_{tissue}(t) = {K}_{trans}.{C}_{AIF} \otimes f(t) $$

### Patlak model

The Patlak model can be seen as a special case of the extended Tofts model. It assumes that F_p_ ≈ ∞ and backflux from the interstitial space is negligible. Two parameters, v_p_ and K_trans_ are estimated using:

D13 $$ {C}_{tissue}(t) = {K}_{trans}{\displaystyle \underset{0}{\overset{t}{\int }}}{C}_{AIF}\left(\tau \right)d\tau + {v}_p.{C}_{AIF}(t) $$

A linearized version of this equation can be obtained by dividing both sides by C_AIF_(t). In our current implementation, v_p_ and K_trans_ estimates using this linear fit provides the starting values for non-linear curve fitting using equation D16.

### The shutter-speed model

The models described above assumes that water, the altered relaxation of which is the actual contributor to the signal changes observed in DCE-MRI, exchanges between different compartments (plasma, interstitium, intracellular space) infinitely fast (referred to as the fast-exchange limit FXL). Studies have shown that the assumption of FXL may underestimate v_e_ and K_trans_ [51, 52]. Springer et al. introduced the shutter-speed model to include the effects of equilibrium inter-compartmental water exchange [53]. The first-generation fast-exchange regime (FXR) shutter-speed model is implemented here. Three parameters, v_e_, K_trans_ and τ_i_ (the average intracellular water lifetime of a water molecule), are estimated using [54]:

D14 $$ {R}_1(t) = \frac{1}{2}\left[2.{R}_{1i} + {r}_1{C}_0(t) + \frac{R_{10}-{R}_{1i}+\frac{1}{\tau_i}}{p_0} - \sqrt{{\left(\frac{2}{\tau_i}-{r}_1{C}_0(t)-\frac{R_{10}-{R}_{1i}+\frac{1}{\tau_i}}{p_0}\right)}^2+\frac{4.\left(1-{p}_0\right)}{\tau_i^2{p}_0}}\right] $$

where

D15 $$ {C}_0(t) = {K}_{trans}{\displaystyle \underset{0}{\overset{t}{\int }}}{C}_{AIF}(t).\ {e}^{-\frac{K_{trans}\left(t-\tau \right)}{v_e}}d\tau, \kern0.37em {p}_0 = \frac{v_e}{f_w}, $$

R_1i_ is the R1 relaxation rate in the intracellular space (defined to be equal to T1^−1^ here) and f_w_ is the fraction of water that is accessible to the contrast agent (user-defined).
